# Pharmacological effects and mechanisms of YiYiFuZi powder in chronic heart disease revealed by metabolomics and network pharmacology

**DOI:** 10.3389/fmolb.2023.1203208

**Published:** 2023-06-23

**Authors:** Yuming Wang, Xue Li, Min Qi, Xiaokai Li, Fangfang Zhang, Yuyu Wang, Junke Wu, Lexin Shu, Simiao Fan, Yunfei Li, Yubo Li

**Affiliations:** ^1^ School of Chinese Materia, Tianjin University of Traditional Chinese Medicine, Tianjin, China; ^2^ TIPRHUYA Advancing Innovative Medicines Ltd., Tianjin, China

**Keywords:** metabolomics, biomarker, chronic heart disease, YiYiFuZi powder, pathways

## Abstract

**Introduction:** YiYiFuZi powder (YYFZ) is a classical formula in Chinese medicine, which is commonly used clinically for the treatment of Chronic Heart Disease (CHD), but it’s pharmacological effects and mechanism of action are currently unclear.

**Methods:** An adriamycin-induced CHD model rat was established to evaluate the pharmacological effects of YYFZ on CHD by the results of inflammatory factor level, histopathology and echocardiography. Metabolomic studies were performed on rat plasma using UPLC-Q-TOF/MS to screen biomarkers and enrich metabolic pathways; network pharmacology analysis was also performed to obtain the potential targets and pathways of YYFZ for the treatment of CHD.

**Results:** The results showed that YYFZ significantly reduced the levels of TNF-*α* and BNP in the serum of rats, alleviated the disorder of cardiomyocyte arrangement and inflammatory cell infiltration, and improved the cardiac function of rats with CHD. The metabolomic analysis identified a total of 19 metabolites, related to amino acid metabolism, fatty acid metabolism, and other metabolic pathways. Network pharmacology showed that YYFZ acts through PI3K/Akt signaling pathway, MAPK signaling pathway and Ras signaling pathway.

**Discussion:** YYFZ treatment of CHD modulates blood metabolic pattern and several protein phosphorylation cascades but importance specific changes for therapeutic effect require further studies.

## 1 Introduction

Chronic heart disease (CHD) is a complex clinical syndrome in which structural or functional abnormalities of the heart cause a range of symptoms of diastolic and systolic dysfunction, such as dyspnea, ankle swelling, and fatigue. CHD is the end-stage of heart disease and a major cause of death from cardiovascular disease (Tsutsui, 2022). The main drugs used to treat heart failure are diuretics, angiotensin-converting enzyme (ACE) inhibitors, angiotensin receptor blockers (ARBs), *β*-blockers, and salt corticosteroid receptor antagonists (MRAs), but the morbidity and mortality rates of the disease remain high (Roger, 2021). Traditional Chinese medicine (TCM) has been used for thousands of years for the treatment of CHD with good results and multi-target advantages, and many studies are showing the use of TCM for the improvement of CHD, which makes the clinical application of TCM in the treatment of CHD possible (Du et al., 2021; Leung et al., 2021; Meng et al., 2021; Liao et al., 2022; Liu et al., 2022).

In Chinese medicine, CHD belongs to the category of “heart paralysis” and “chest paralysis,” and the main pathogenesis of CHD is a deficiency of positive energy and paralysis by cold and dampness. This formula is from the book “Jin Gui Yao Lve.” Coix seed has multiple pharmacological effects such as anti-tumor, improving body immunity, hypoglycemia, anti-inflammatory and analgesic, and regulating lipid metabolism, and its important pharmacological activities in the treatment of cervical cancer, lung cancer, and gastrointestinal tract tumors have been confirmed in clinical practice (Ni et al., 2021; Zhou et al., 2021; Sui and Xu, 2022; Yang et al., 2022). In a related experimental study, Coix seed was found to improve Th1/Th2 cytokines in mice to restore immune homeostasis after administration (Wang H. et al., 2022). Fuzi is commonly used in clinical practice for the treatment of heart diseases such as heart failure, and its main chemical constituents are alkaloids with cardiac analgesic, anti-myocardial ischemic, anti-arrhythmic, and improving the energy metabolism of cardiomyocytes (Zhang et al., 2017; Yan et al., 2020; Chen et al., 2022; Tai et al., 2022).

Metabolomics and network pharmacology are effective tools to elucidate the potential mechanisms of TCM compounding. In recent years, metabolomics has been widely used in biomedical research, and its target is endogenous metabolites with small molecular weight in the organism, and by detecting the changes of endogenous metabolites after the disturbance of the organism, the differential metabolites associated with the disturbance can be identified, and the metabolic pathways can be elucidated in combination with bioinformatics analysis, and then the biological metabolic mechanism can be analyzed (Li et al., 2020; Cui et al., 2021; Nicholson, 2021; Sun C. et al., 2021; Buergel et al., 2022; Santos-Gallego et al., 2022). Network pharmacology is a joint application of bioinformatics, systems biology and multidirectional pharmacology to study drugs. The “multi-target” and “synergistic mechanism” emphasized by cyber pharmacology are in line with the “holistic concept” and “diagnosis and treatment” emphasized by TCM theory. “Through the analysis of genes, proteins, diseases, drugs and other real data obtained from databases and experiments, the intrinsic relationship between multi-component and multi-target effects of TCM on the body can be explored from a systematic and holistic perspective, which is important for explaining the potential mechanisms of TCM treatment (Li et al., 2019; Yao et al., 2020; Li et al., 2021; Shi et al., 2022; Zhou et al., 2022). The combination of network pharmacology and metabolomics can complement each other to reveal the biological significance and mechanism of drug action more comprehensively.

In this study, we first established a rat model of CHD suitable for testing of pharmacological effects and metabolomic analysis on plasma samples to reveal potential biomarkers and metabolic pathways; secondly, we performed network pharmacological analysis on the *in vivo* components of YYFZ to predict the targets and pathways of action as well as allowing the in-depth study of the material basis and mechanism of action of YYFZ in the treatment of CHD.

## 2 Materials and methods

### 2.1 Instruments and reagents

Adriamycin hydrochloride was purchased from Solarbio (China). Purified water was purchased from Watson’s (China). Isoflurane was purchased from Rwd Life Science Co., Ltd. (China). Saline for injection was purchased from SSY Group Limited. Captopril was purchased from Shanghai Xudong Haipu Pharmaceutical Co., Ltd. (China). Coix Seed was purchased from Hunan Yaoshengtang Traditional Chinese Medicine Technology Co., Ltd. (China). Fuzi was purchased from Hebei Meiwei Pharmaceutical Co., Ltd. (China).

Vevo small animal ultrasound imager was purchased from Visualsonics Co., Ltd. (United States). ALLLEGRATM-64R high-speed frozen centrifuge was purchased from Beckman Co., Ltd. (United States). TecanInfiniteF50 enzyme labeler was purchased from Tecan, Co., Ltd. (Switzerland). UPLC/Q-TOF/MS was purchased from Waters Corporation Co., Ltd. (United States). The rat TNF-*α* kit and rat BNP kit were purchased from Nanjing Jiancheng Biotechnology Co., Ltd. (China). The pathological sections of heart tissue were processed by Hunan Fenghui Biotechnology Co., Ltd. (China).

### 2.2 Preparation of YYFZ decoction

Weighed coix seeds and Fuzi, mixed in the ratio of 5:3, placed in a round bottom flask, added 10 times the amount of water and soak for 45 min. The extract was then extracted by reflux extraction method for 1 h. The extract was filtered through 3 layers of gauze. The remaining drug residue was added with 8 times the amount of water and extracted for 45 min. The two filtrates were combined and concentrated to a viscous infusion (concentration of 1 g/mL in terms of raw drug). The samples were stored in a refrigerator at 4°C and set aside.

### 2.3 Animals

Sixty SPF-grade male Wistar rats (190–220 g, purchased from Beijing Spelford Biotechnology Co., Ltd., license number: SCXK (Beijing) 2019–0010) were selected, and the rats were acclimatized and fed at a temperature of 20°C–26°C, relative humidity of 40%–70%, ventilation of 10–15 times/h, and light of 12 h light/dark for 7 days, and all animals The feeding process was carried out by the operating procedures for clean grade laboratory animals. The study was approved by the Animal Ethics Committee of Tianjin University of Traditional Chinese Medicine (TCM-LAEC2021241), and ethical norms were followed in handling animals during the experiment to minimize animal numbers and suffering.

### 2.4 Establishment of CHD rat models and grouping

After 1 week of acclimatization feeding, 10 rats were randomly selected as the NS group (control group), and the remaining rats were injected intraperitoneally with 1.25 mg/mL aqueous solution of adriamycin hydrochloride at a dose of 1.25 mg/kg twice a week for 8 weeks, with a cumulative dose of 18 mg/kg; the NS group was injected with the same dose of saline. After the end of modeling, the rats were randomly divided into 5 groups: Model group, Captopril group, YYFZ-H group, YYFZ-M group, YYFZ-L group, 10 rats in each group and treated according to the dosing. YYFZ-H, YYFZ-M, and YYFZ-L groups were treated with 5.25 g/kg, 2.63 g/kg and 1.31 g/kg by gavage. The Captopril group was given 6.75 mg/kg by gavage. The NS and Model groups were given the same volume of pure water as a control and administered once a day for a treatment period of 30 days.

### 2.5 Observation and detection of indicators

#### 2.5.1 General morphological observation

From the beginning of the modeling, the experimental animals were closely observed and recorded for weight, coat condition, mental status, respiratory system condition and survival.

#### 2.5.2 Evaluation of rat CHD model

At the end of the modeling, all rats fasted for 12 h. Ten rats were randomly selected from the NS group and the model group, anesthetized with isoflurane. Cardiac function was measured using a vevo small animal ultrasound imager, with cardiac ejection fraction (EF) less than 55% as the modeling criterion. At the same time, blood was taken from the inner canthus of the rat’s eye, placed at room temperature for 30 min, and then centrifuged for 10 min at 4°C and 3,500 rpm/min in a freezing centrifuge for the detection of BNP and TNF-*α*.

#### 2.5.3 Detection of cardiac function indexes

The rats were anesthetized with a VMR small animal respiratory anesthesia machine, skin was prepared on the chest using hair removal cream, coupling agent was applied, and an ultrasound probe was used to perform ultrasound examination of the rat’s heart from the long axis of the parasternal bone. In M ultrasound mode, and the rat’s EF, fractional shortening (FS), left ventricular posterior wall end-systolic thickness (LVPW; s), left ventricular posterior wall end-diastolic thickness (LVPW; d), left ventricular end-systolic internal diameter (LVID; s), left ventricular internal diameter end-diastolic internal diameter (LVID; d), left ventricular end-systolic septal thickness (IVS; s), Left ventricular end-diastolic septal thickness (IVS; d), and the average of 3 cardiac cycles were taken for each sample.

### 2.6 Observation and detection of indicators

At the end of day 30 dosing, all rats fasted without water for 12 h. After anesthetizing the rats, the cardiac function indexes were first tested using VMR, and then subsequent operations were performed under anesthesia.

#### 2.6.1 Collection of blood samples

Blood was collected from rats through the abdominal aorta to the maximum extent possible, and half of the whole blood samples were placed in heparin anticoagulation tubes and the other half in EP tubes, which were left for 30 min and then pretreated. The plasma samples in the anticoagulation tubes were centrifuged at 4°C, 3,500 rpm for 10 min, and the supernatant was frozen in a −80°C refrigerator for subsequent metabolomic analysis; the blood samples in the EP tubes were centrifuged at 4°C, 3,500 rpm for 10 min, and the supernatant was frozen in a −80°C refrigerator for subsequent serum biochemical analysis.

#### 2.6.2 Heart index

The hearts of rats were removed and weighed after blood sampling from the abdominal aorta, and the organ index was calculated: organ index = organ (g)/body weight (kg).

#### 2.6.3 Detection of serum biochemical indexes

The levels of BNP and TNF-*α* in rat serum were measured by ELISA kits.

#### 2.6.4 Cardiac histopathology

The rat heart was taken and placed in a 4% tissue fixating solution for 24 h, then dehydrated, paraffin-embedded wax blocks were made, which were successively sliced, dissected, baked, and stained by HE. Morphological changes in rat cardiomyocytes were observed under a light microscope.

### 2.7 Metabolomics analysis

#### 2.7.1 Sample preparation

Frozen rat plasma samples were removed from the −80°C refrigerator and thawed first in a 4°C refrigerator. 100 μL of plasma was pipetted from the samples, 300 μL of chromatographically pure acetonitrile was added at a volume ratio of 1:3, vortexed and mixed for 5 min, then centrifuged in a refrigerated centrifuge at 13,000 rpm/min for 15 min, and the supernatant was aspirated for UPLC-Q-TOF/MS analysis.

#### 2.7.2 Preparation of QC sample

10 μL of each plasma sample was taken in an EP tube, vortexed for 1 min, and then centrifuged at 4°C at 13,200*g for 15 min, and the supernatant was aspirated for methodological investigation.

#### 2.7.3 Instrument conditions

In this experiment, UPLC-Q-TOF/MS was used to characterize metabolites. UPLC separation was performed on an ACQUITY UPLC BEH C_18_ column (2.1 mm × 100 mm, 1.7 μm, Waters Co., United States) at 45°C. The mobile phase consists of water (A) and acetonitrile (B) (both containing 0.1% formic acid). The gradient elution procedure is as follows: 0–0.5 min, 1%B; 0.5–2 min, 1%-50%B; 2–9 min, 50%-99%B; 9–10 min, 99%B; 10–10.5 min, 99%-1%B; 10.5–12 min, 1%B, the flow rate was 0.3 mL/min. The injection volume was 5 μL.

After separation, mass spectra were detected and analyzed using an electrospray ion source (ESI) in positive and negative ionization modes. Ion source parameters are set as follows: The capillary voltage is 3.0 kV, the drying gas temperature is 325°C, the atomized gas pressure is 310 kPa, the drying gas flow is 0.26 mL/min, the desorption gas flow is 600 L/h, the source temperature is 120°C, the desorption temperature is 350°C, and the cone gas flow is 50 L/h.

#### 2.7.4 Data processing

Raw data were exported by Masslynx4.1 (Waters, United States) software, after which the data were imported into SIMCA 14.1 statistical software (Umetrics Corporation, Sweden) for multivariate statistical analysis. Subsequently, SPSS 26.0 was applied for statistical tests and the appropriate test was selected to determine whether metabolites changed significantly (*p* < 0.05) in the statistical analysis. The metabolites screened by the above analysis are input into the MetaboAnalyst platform(https://www.metaboanalyst.ca/) for cluster analysis and Mate-Pa analysis.

### 2.8 Network pharmacology

#### 2.8.1 Blood-inlet-components in YYFZ

Based on the incoming components of YYFZ identified in our previous study (Supplementary Material), the information on these components was retrieved in TCMSP (http://lsp.nwsuaf.edu.cn/tcmsp.php) for relevant target information. On the other hand, the molecular structure formula was drawn in Chemdraw, saved in.sdf format and uploaded to PharmaMapper (http://lilab-ecust.cn/pharmmapper/index.html) for predicting potential targets, and SwissTargetPrediction (http://www.swisstargetprediction.ch/) for predicting the results. As a supplement, the targets of all components were combined and de-weighted after conversion to Genename by UniProt (https://www.UniProt.org/) (UniProt Consortium, 2013).

#### 2.8.2 Establishment of CHD disease target library

Search by Genecards (https://www.genecards.org/), TTD data (http://bidd.nus.edu.sg/group/cjttd/) (Wang et al., 2020), OMIM (https://omim.org/) (Amberger and Hamosh, 2017) databases The targets related to the pathogenesis of “chronic heart disease” were combined by removing duplicate targets from several databases.

#### 2.8.3 Visualization and analysis of active ingredient-target-disease network

PPI (protein-protein interaction) maps were created through protein interaction analysis via STRING database and imported into Cytoscape 3.8.2 for intra-network visualization of PPI data between potential therapeutic targets of diseases and mapping of active ingredient-target-disease networks.

KEGG pathway enrichment analysis was performed on the network nodes using DAVID database to obtain the functional pathways involved in disease modulation by each component-acting target of YYFZ bulk, and the enriched pathways were visualized using the advanced bubble map function of the Omicshare platform.

## 3 Results

### 3.1 Pharmacodynamic study of YYFZ on CHD rat model

#### 3.1.1 General conditions of rats

During the modeling of CHD rats, the rats in the NS group grew naturally, fed and drank normally, and were in good condition, and there was no death. During the modeling process, the rats’ body weight growth slowed down significantly, and they lost their body weight and hair. After the fourth week, the rats gradually appeared ascites, abdominal distension, panting and shortness of breath, and individual rats lost their hair in flakes; until the seventh week, the rats had obvious abdominal distension, diarrhea, watery stool, filthy perianal area, shortness of breath, vertical hair, loose fur, lack of blood in the feet and paws and ears, mental inactivity, and increased secretion around the eyes and nose. After the start of administration, the condition of the rats in the Captopril group, YYFZ-H, YYFZ-M, and YYFZ-L improved, while no improvement was observed in the Model group. In addition, hepatomegaly was seen in the Model group at autopsy, and some of them had renal edema with mesenteric adhesions.

#### 3.1.2 Weight gain of rats

**FIGURE 1:**
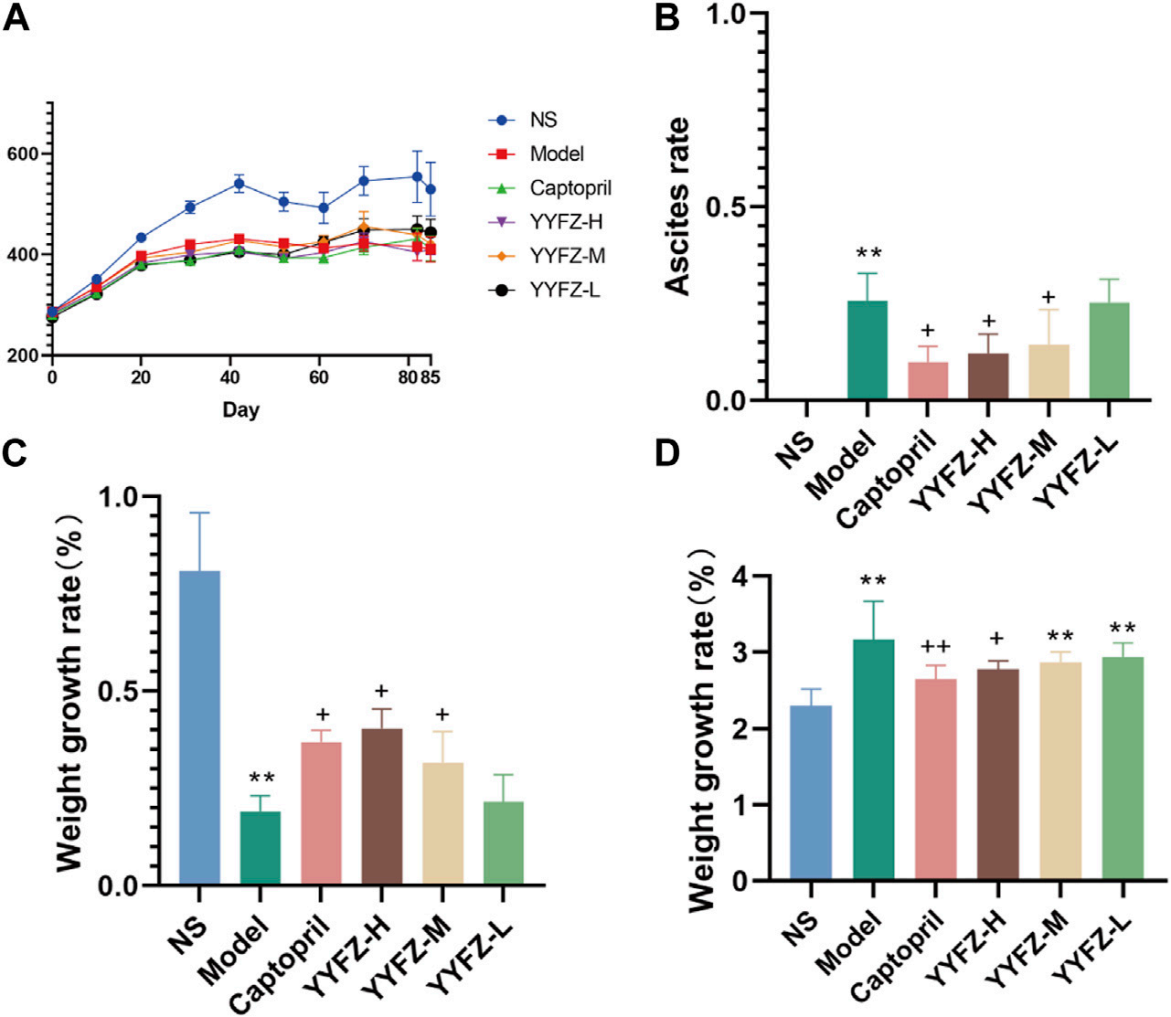
**(A)** Weight change trend of rats; **(B)** Ascites of rats in each group; **(C)** Weight growth of rats in each group; **(D)** Heart index of rats in each group.

After the beginning of modeling, it can be seen from (Figure 1A) that the weight gain of rats in the model group slowed down significantly, and there was a significant difference in body weight after the second injection (*p* < 0.01) until the end of the experiment. During the modeling period, the body weight of the NS group was stable, while that of the model group was slow, and occasionally fluctuated in the later period, but there was almost no obvious increase. The rats in the administration group showed no significant difference in body weight during the modeling period compared to NS (*p* < 0.05) and showed a significant increase after administration (*p* < 0.05). At the late stage of the experiment, the weight changes of rats were difficult to directly reflect the drug effect because of the severe abdominal ascites of some rats. Therefore, in this study, the therapeutic effect of the drug was reflected by calculating the body weight growth rate of rats, and the body weight growth rate % = (body weight without ascites—body weight before modeling)/body weight before modeling * 100%. It can be seen from Figure 1C that the body weight growth rate of the Model group and drug administration group was significantly different from that of the NS group (*p* < 0.05), while compared with the Model group, the body weight of Captopril group, YYFZ-H group and YYFZ-M group was significantly increased (*p* < 0.05).

(Compared with NS group, *n* = 10, ‾x ± SD ^*^: *p*<0.05, ^**^: *p* < 0.01; compared with Model group, ^+^: *p* < 0.05, ^++^: *p* < 0.01)

#### 3.1.3 Ascites in rats

At the late stage of modeling, some rats showed significant signs of ascites; the ascites condition of the rats in the administered group did not continue to worsen as the administration treatment began. As seen in Figure 1B, the ascites of rats in Captopril, YYFZ-H and YYFZ-M groups appeared significantly different from the Model group (*p*< 0.05), and there was no significant difference in the low-dose group, suggesting that Captopril and high and medium doses of YYFZ have better efficacy.

#### 3.1.4 Rat heart function

At the end of modeling, to determine the success of the model, we performed cardiac function tests on the rats. As seen in Table 1, the EF and FS in the Model group were significantly lower than those in the NS group (*p* < 0.05), indicating the success of the model. The rats were grouped after modeling, and cardiac function tests were performed after grouping. As seen in Table 2, compared with the NS group, LVPW; s and LVPW; d was significantly lower in the Model and drug administration groups (*p* < 0.05), with LVPW; d significantly higher in the Captopril group than in the Model group (*p* < 0.05). IVSs and IVSd in the Model group and YYFZ-H, YYFZ-M and YYFZ-L groups were significantly lower than those in the NS group (*p* < 0.05), with significant differences between IVSs in the YYFZ-H and YYFZ-M groups and the Model group (*p* < 0.05). The IVSs and IVSd in the Captopril group were not statistically different from those in the NS group and were significantly higher than those in the Model group (*p* < 0.05).

**TABLE 1:** Comparison of cardiac structural and functional indices between modeled rats and blank rats.

Indicators	NS	Model
EF(%)	67.12 ± 1.59	49.23 ± 1.54^**^
FS(%)	38.53 ± 1.24	25.79 ± 0.98^**^
LVPW; s (mm)	2.95 ± 0.14	2.45 ± 0.14^*^
LVPW; d (mm)	1.96 ± 0.11	2.19 ± 0.15
LVID; s (mm)	4.52 ± 0.18	5.38 ± 0.23^*^
LVID; d (mm)	7.36 ± 0.31	7.27 ± 0.33
IVS; s (mm)	2.93 ± 0.15	2.92 ± 0.19
IVS; d (mm)	1.99 ± 0.15	2.14 ± 0.15

^*^ *p* < 0.05, ^**^ *p* <0.01.

**TABLE 2:** Cardiac indexes (LVPW;s, LVPW;d, IVSs, IVSd) of rats in each group before drug administration.

Group	LVPW;s(mm)	LVPW;d(mm)	IVSs(mm)	IVSd(mm)
NS	3.68 ± 0.17	2.46 ± 0.08	3.30 ± 0.10	2.31 ± 0.09
Model	2.28 ± 0.11^**^	1.73 ± 0.10^**^	2.48 ± 0.12^**^	1.84 ± 0.09^**^
Captoril	2.63 ± 0.08^**^	2.12 ± 0.09^**^^+^	2.85 ± 0.19^+^	2.19 ± 0.13^+^
YYFZ-H	2.60 ± 0.14^**^	2.04 ± 0.11^**^	2.81 ± 0.14^*+^	2.04 ± 0.06^*^
YYFZ-M	2.44 ± 0.09^**^	1.98 ± 0.07^**^	2.68 ± 0.10^**+^	1.94 ± 0.06^**-^
YYFZ-L	2.32 ± 0.13^**^	1.84 ± 0.06^**-^	2.58 ± 0.13^**^	1.88 ± 0.13^**-^

Compared to the NS group.^*^ *p* < 0.05, ^**^ *p* < 0.01; Compared with the Model group. ^+^ *p*<0.05, ^++^ *p* < 0.01; Compared with the Captoptil group, Compared with the Captoptil group, ^–^ *p* < 0.05, ^––^ *p* < 0.01.

**FIGURE 2:**
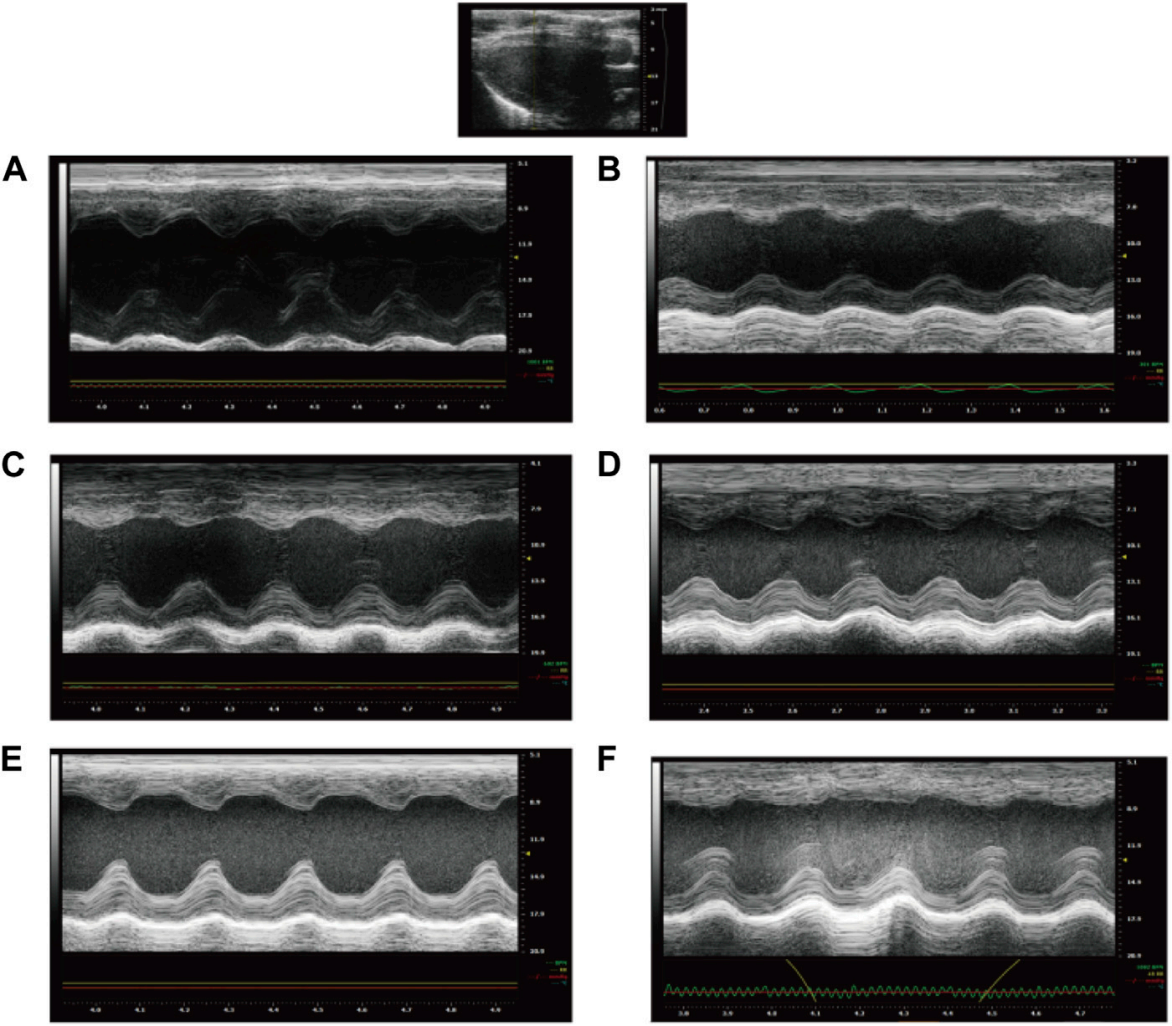
Echocardiography of rats after treatment (**(A)**: NS group; **(B)** Model group; **(C)** Captopril group; **(D)**YYFZ-H group; **(E)** YYFZ-M group; **(F)** YYFZ-L group).

Cardiac function tests were performed on the rats at the end of the dosing. As shown in Table 3 and Figure 2. The EF and FS in the Model group were significantly lower than those in the NS group (*p* < 0.05), indicating that the systolic-diastolic function of the heart was reduced in the Model group. The EF and FS in the Captopril and YYFZ-H groups were significantly higher than those in the Model group. Compared with the NS group, LVID;d was significantly lower (*p* < 0.05) and LVID;s was significantly higher (*p* < 0.05) in the Model group. LVID;d, LVID;s in the YYFZ-M and YYFZ-L groups were significantly lower than those in the NS and Model groups (*p* < 0.05). LVID;d, LVID;s in the Captopril and YYFZ-H groups were not significantly different from the NS group, but were significantly different from the Model group (*p* < 0.05).

**TABLE 3:** Comparison of cardiac function indices (FS, EF, LVID;s, LVID;d) in rats in each group after drug administration.

Group	FS(%)	EF(%)	LVID;s(mm)	LVID;d(mm)
NS	44.96 ± 2.08	72.61 ± 2.13	4.60 ± 0.14	8.45 ± 0.14
Model	26.64 ± 0.86^**^	48.51 ± 1.93^**^	5.58 ± 0.11^**^	6.89 ± 0.11^**^
Captopril	35.52 ± 1.59^**++^	59.29 ± 2.61^**++^	4.98 ± 0.18^+^	7.8 ± 0.25^++^
YYFZ-H	33.11 ± 1.74^**++^	56.42 ± 0.99^**++^	5.01 ± 0.13^++^	8.09 ± 0.16^++^
YYFZ-M	30.35 ± 1.73^**^	53.18 ± 2.16^**^	5.24 ± 0.15^**^	7.7 ± 0.19^**++^
YYFZ-L	28.95 ± 1.91^**^	51.96 ± 3.00^**^	5.42 ± 0.19^**^	7.48 ± 0.17^**++^

Compared to the NS group.^*^ *p* < 0.05, ^**^ *p* < 0.01; Compared with the Model group. ^+^ *p* < 0.05 ,^++^ *p* < 0.01; Compared with the Captoptil group, ^–^ *p* < 0.05, ^––^ *p* < 0.01.

#### 3.1.5 Rat cardiac index

After weighing and comparing, we found that there was no significant difference in the heart weight of the rats in each group, presumably related to the larger size of the rats in the NS group. After conversion into the cardiac index, it can be seen from Figure 1D that the cardiac index of the Model group and each dosing group was significantly higher than that of the NS group (*p* < 0.05), and the mean value of the cardiac index of each dosing group was lower than that of Model group, but only Captopril group and YYFZ-H group were significantly lower than that of Model group (*p* < 0.05), and the difference between the cardiac index of the remaining dosing groups and Model group was not The difference between the cardiac index of the remaining administration groups and the Model group was not statistically significant.

#### 3.1.6 Detection of serum BNP and TNF-*α* in rats

**FIGURE 3:**
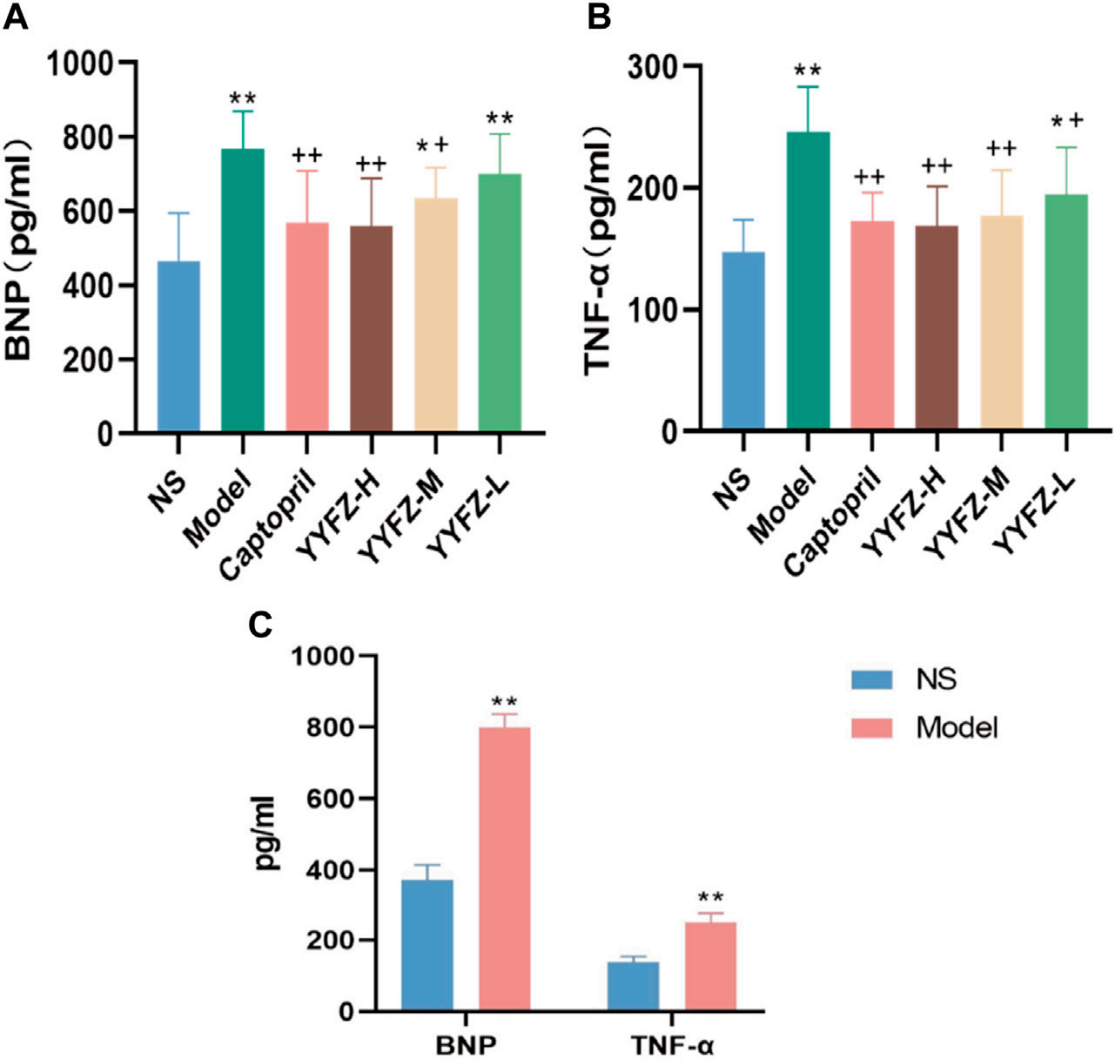
**(A)** Reduction of BNP after treatment; **(B)** Reduction of TNF after treatment; **(C)** BNP and TNF-α before treament.

To assess the rat modeling situation, this study used Elisa kits to detect BNP and TNF-*α* contents in rat serum, as shown in Figure 3C, there was a significant increase in BNP content in the serum of model rats about 2.16 times higher than that of NS group, and the difference was statistically significant (*p* < 0.01); TNF-*α* in model rats was about 1.6 times higher than that of NS rats, and the difference was statistically significant (*p*< 0.01).

After the administration, the serum BNP and TNF-*α* levels in each group are shown in Figures 3A, B. The serum BNP levels in the Model, YYFZ-M and YYFZ-L groups were significantly higher than those in the NS group (*p* < 0.05), with the serum BNP in the YYFZ-M group being significantly lower than that in the Model group (*p* < 0.05) and the YYFZ-L group not significantly different from that in the Model group. The differences between the Captopril and YYFZ-H groups were statistically significantly lower than those in the Model group (*p* < 0.05) and the NS group (*p* < 0.05). The TNF-*α* levels in the Captopril, YYFZ-H and YYFZ-M groups were significantly lower than those in the Model group (*p* < 0.05), and there was no significant difference with the NS group. (Compared with the NS group, *n* = 10, ‾x ± SD ^*^: *p* <0.05, ^**^: *p*<0.01; compared with the Model group, ^+^: *p*<0.05, ^++^: *p*<0.01).

#### 3.1.7 Pathological results of myocardial tissue in rats

**FIGURE 4:**
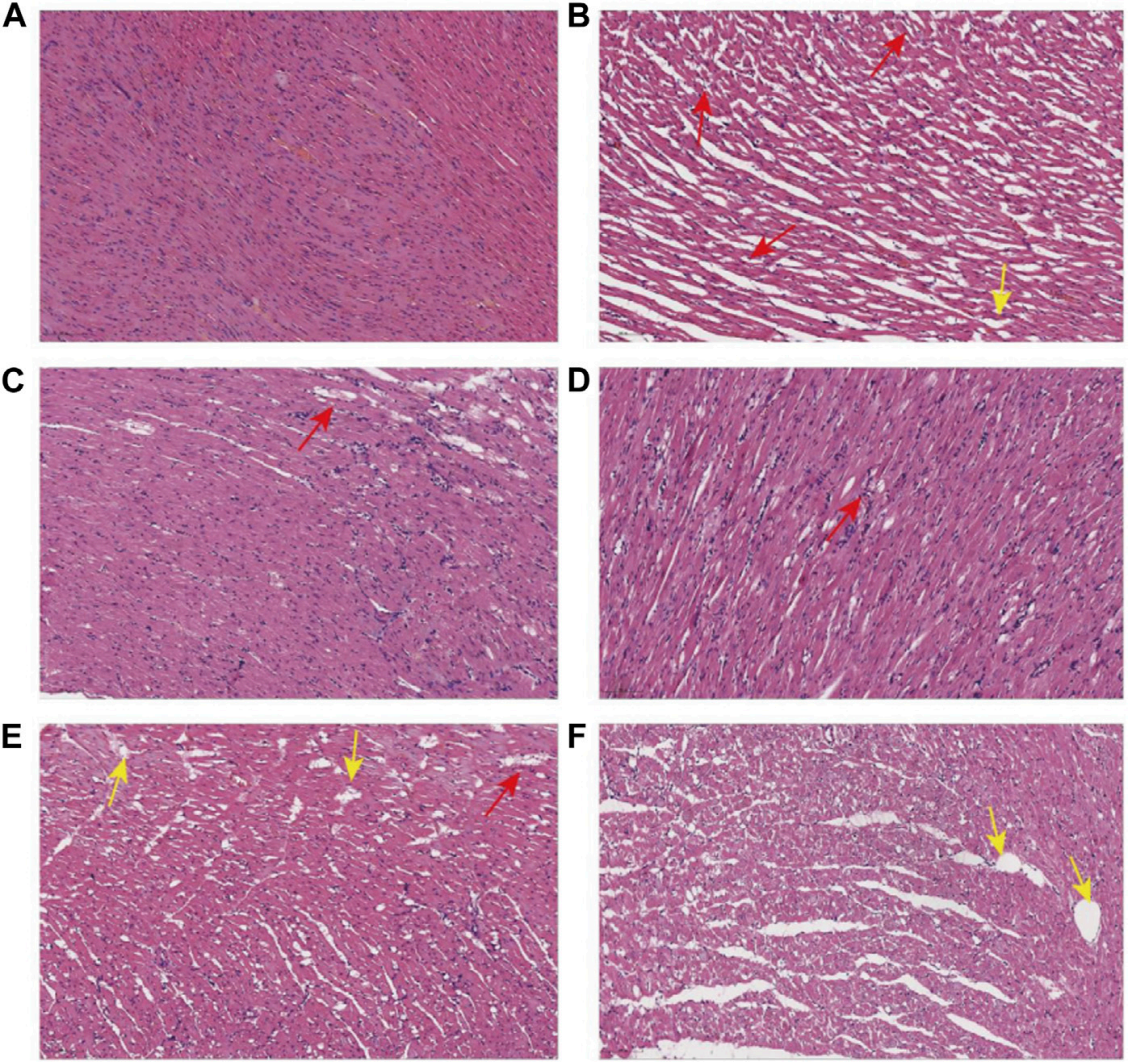
Pathological sections of myocardial tissue of rats in each group (**(A)**: NS group; **(B)** Model group; **(C)** Captopril group; **(D)** YYFZ-H group; **(E)** YYFZ-M group; **(F)** YYFZ-L group).

The results of histopathological HE (hematoxylin-eosin) staining of rat myocardium are shown in Figure 4: In the NS group, myocardial cells were clearly arranged and tightly packed, with a few myocardial transverse breaks, and vascular proliferation and inflammatory cell infiltration were not obvious. The myocardial cells in the Model group were unevenly stained, with relatively blurred transverse lines, reduced volume of some cells, and deepened staining of the envelope; the myocardial interstitium was obviously edematous, with loosely arranged cells and fat vacuoles within the cells (yellow arrows), an increased number of small blood vessels between the myocardium (red arrows), and a small number of lymphocytes between the myocardium. Captopril group and YYFZ-H group: myocardial cell transverse lines were clear, there was a small amount of edema in myocardial interstitium, and the area of lax cell arrangement was reduced compared with the model group. YYFZ-M group and YYFZ-L group: myocardial cell transverse lines were clear, the cell volume was reduced, the arrangement was more laxer, there was edema in the cell interstitium, and the number of blood vessels was increased.

### 3.2 Metabolomics analysis results

#### 3.2.1 Methodological investigation

**FIGURE 5:**
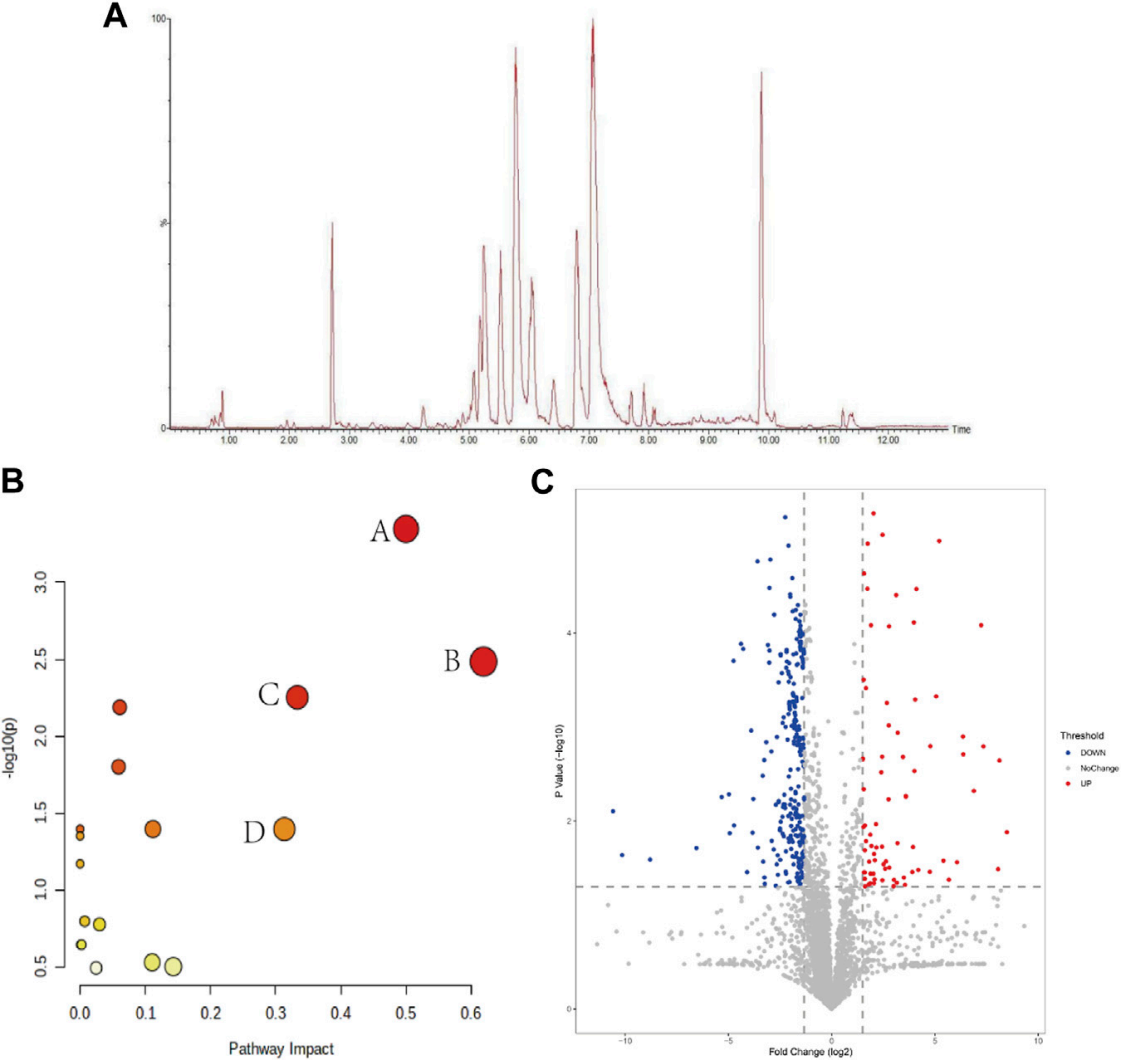
**(A)** BPI plots of QC samples; **(B)** Marker metabolic pathway analysis (A: phenylalanine, tyrosine and tryptophan biosynthesis; B: linolenic acid metabolism; C: phenylalanine metabolism; D: arachidonic acid metabolism); **(C)** intergroup volcano plot analysis between NS and Model groups.

After collecting the data by UPLC-Q-TOF/MS analysis, we randomly selected 20 peaks from the obtained QC sample profiles (as shown in Figure 5A) and calculated the RSD values of their peak areas and retention times. RSD<16.17%, retention time RSD<0.92%; sample stability study in the sample at 0 h, 6 h, 12 h, 18 h, 24 h time points in the peak area RSD<12.45%, retention time RSD<0.59%. The above indicates that the instrument precision, method precision and sample stability are good, indicating that the method is reliable and can be followed up.

#### 3.2.2 Metabolomics data pre-processing and multivariate statistical analysis

The raw data were exported from Masslynx4.1 software, and the data were subjected to a normality test and chi-square test after 80% modification, and suitable methods were selected to compare the NS and Model groups between groups, and *p*-values and fold change (FC) were calculated, and the substances and *p*-values and log2 (FC) values were entered into the Wukong data analysis cloud platform for volcano plot analysis to visually display the NS and Model groups group in the different substances. As shown in Figure 5C, the horizontal coordinate is log2 (FC), the greater the difference the more distant the metabolite is distributed, and the vertical coordinate is the *p*-value, the greater the difference the more distant the substance is from the horizontal axis, so the substance distributed in the upper left right corner is usually considered as a potential difference marker. Each of these points represents data, and the gray color is for substances with no significant changes, while the red and blue colors are for substances with significant elevated changes, which are potential differential substances that we need to pay attention to in the subsequent processing.

**FIGURE 6:**
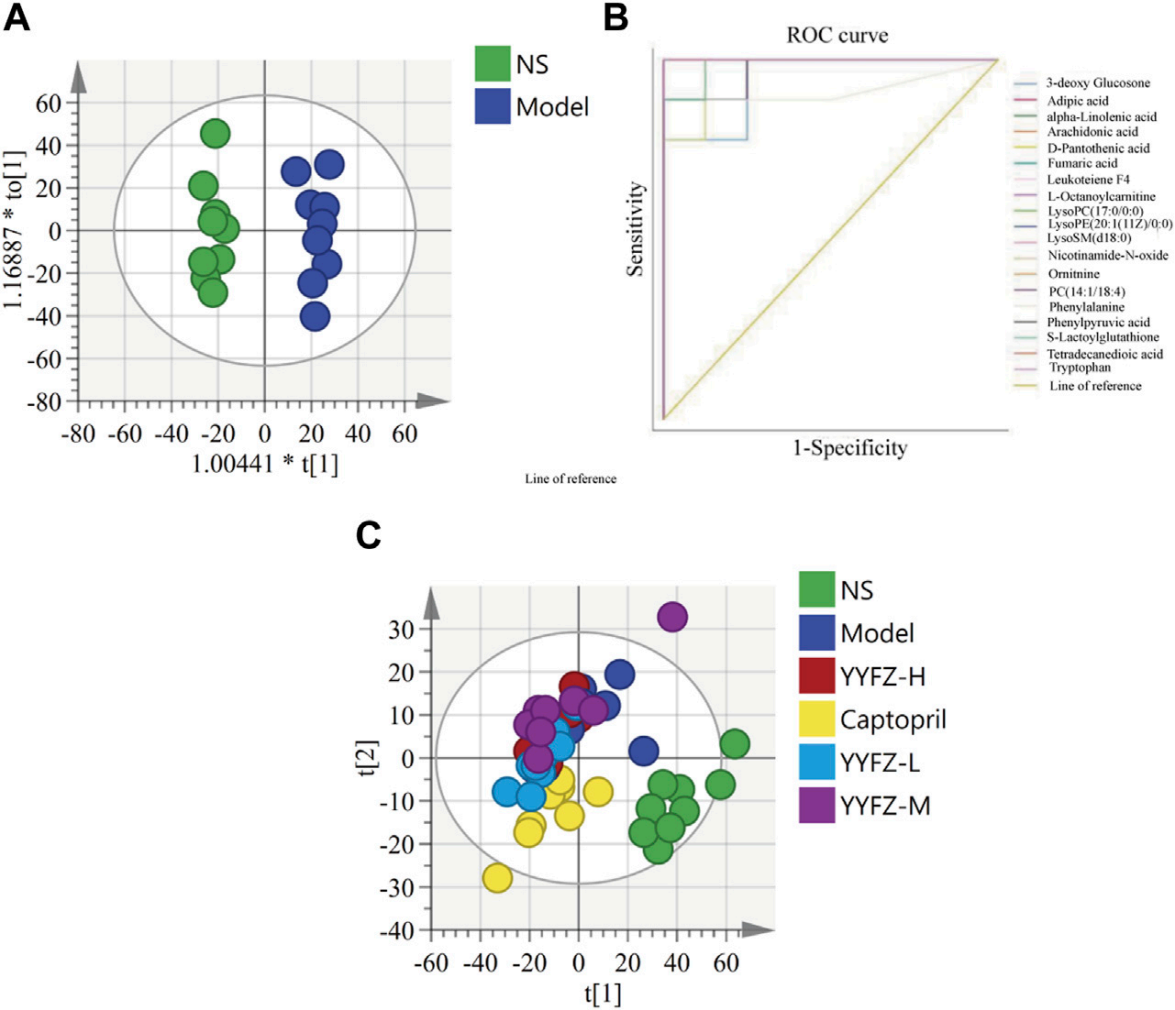
**(A)** OPLS-DA; **(B)** CHD marker ROC analysis **(C)** PCA.

Multivariate statistical analysis was used to discriminate the changes of metabolites within the plasma of healthy and model rats. For this, we first set up an unsupervised PCA model as shown in Figure 6C. Each point in the figure represents a sample, respectively, and the color is used to distinguish the rat groups, and the farther the distance between the points of different groups indicates the greater difference. We can see that there is a certain convergence in the distribution of NS and Model groups, and Model and drug administration groups in the PCA model. Therefore, to further differentiate, we further established a supervised OPLS-DA model (Figure 6A), and we can see that the NS group and Model group are distributed on both sides in the OPLS-DA model, with obvious distinction.

#### 3.2.3 Identification of CHD biomarkers

The SPSS26.0 software was used to test for normality and the chi-square test for the differential substances. *t*-test and Mann-Whitney *u*-test were selected to test for significance according to whether the data distribution was normal and chi-square, and the differential substances with significant changes (*p* < 0.05) were considered as differential markers of heterogeneous disease and treatment. Finally, we obtained 732 NS-CHD differential substances, respectively. We entered m/z values in the HMDB database to search, selected [M + H]^+^, [M + K]^+^, [M + Na]^+^ in ion addition mode, and identified metabolites by MS/MS analysis, metabolite database information, and literature information. We finally identified 19 markers, which were leukotriene F4, arachidonic acid, lysoSM(d18:0), tryptophan, phenylpyruvic acid, S-lactoylglutathione, phenylalanine, ornithine, nicotinamide-N-oxide, fumaric acid, adipic acid, D-pantothenic acid, PC(14:1/18:4), tetradecanedioic acid, 3-deoxy glucosone, *α*-linolenic acid, L-octanoylcarnitine, lysoPE (20:1(11Z)/0:0), lysoPC (17:0/0:0), see Table 4 for specific information. 16 markers were upregulated and 3 markers were downregulated in the Model group.

**TABLE 4:** Differential metabolite information.

No.	tR/min	Metabolite	Formula	Parent ion	Theoretical value	Measured value	ppm	Model/NS	Captopril/Model	YYFZ-H/Model	YYFZ-M/Model	YYFZ-L/Model
1	5.47	Leukoteiene F4	C_28_H_44_N_2_O_8_S	M + K	607.2416	607.2443	4.45	↓^**^	↑^*^	↑^*^	↑^*^	↑
2	6.6	Arachidonic acid	C_20_H_32_O_2_	M + K	343.2006	343.2029	6.70	↑^**^	↓^**^	↓^*^	↓^*^	↓
3	4.59	LysoSM(d18:0)	C_23_H_51_N_2_O_5_P	M + K	505.3174	505.3201	5.34	↑^**^	↓^*^	↓^**^	↓^*^	↓^*^
4	0.91	Tryptophan	C_11_H_12_N_2_O_2_	M + Na	227.0796	227.0784	−5.28	↑^**^	↓^*^	↓^*^	↓^*^	↓
5	0.89	Phenylpyruvic acid	C_9_H_8_O_3_	M + H	165.0552	165.0547	−3.02	↓^**^	↑^**^	↑^*^	↑	↑
6	8.09	S-Lactoylglutathione	C_13_H_21_N_3_O_8_S	M + H	379.1049	379.1054	1.32	↑^**^	↓^**^	↓^**^	↓^*^	↓^*^
7	1.72	Phenylalanine	C_9_H_11_NO_2_	M + H	166.0827	166.0831	2.41	↑^**^	↓^*^	↓^**^	↓^*^	↓^*^
8	3.09	Ornithine	C_5_H_12_N_2_O_2_	M + H	133.0991	133.0968	−17.28	↑^**^	↓^*^	↓^*^	↓^*^	↓
9	0.84	Nicotinamide-N- oxide	C_6_H_6_N_2_O_2_	M + Na	161.0359	161.0376	10.56	↑^**^	↓^**^	↓^**^	↓^*^	↓
10	6.79	Fumaric acid	C_4_H_4_O_4_	M + Na	139.0007	139.0029	15.83	↑^**^	↓^**^	↓^*^	↓^*^	↓^*^
11	2.23	Adipic acid	C_6_H_10_O_4_	M + H	147.0657	147.0636	−14.28	↑^**^	↓^*^	↓^**^	↓^*^	↓^*^
12	1.71	D-Pantothenic acid	C_9_H_17_NO_5_	M + H	220.1144	220.1153	4.09	↑^**^	↓^*^	↓^*^	↓	↓
13	5.48	PC(14:1/18:4)	C_40_H_70_NO_8_P	M + K	762.4469	762.4483	1.84	↑^**^	↓^**^	↓^**^	↓^*^	↓
14	6.06	Tetradecanedioic acid	C_14_H_26_O_4_	M + K	297.1468	297.1501	11.11	↓^**^	↑^*^	↑^**^	↑^*^	↑^*^
15	5.89	3-deoxy Glucosone	C_27_H_42_O_11_	M + Na	565.2661	565.2638	−4.69	↑^**^	↓^*^	↓^*^	↓^*^	↓
16	5.66	*α*-Linolenic acid	C_18_H_30_O_2_	M + H	279.2324	279.2361	13.25	↑^**^	↓^**^	↓^**^	↓^*^	↓^*^
17	2.82	L-Octanoylcarnitine	C_15_H_29_NO_4_	M + H	288.2169	288.2142	−9.37	↑^**^	↓^*^	↓^*^	↓^*^	↓
18	5.09	LysoPE(20:1(11Z)/0:0)	C_25_H_50_NO_7_P	M + Na	530.3216	530.3247	5.84	↑^**^	↓^*^	↓^*^	↓^*^	↓^*^
19	6.11	LysoPC(17:0/0:0)	C_25_H_52_NO_7_P	M + H	510.3641	510.3567	−14.5	↑^**^	↓^*^	↓^**^	↓^*^	↓

*p<0.05. **p<0.01.

#### 3.2.4 Hierarchical clustering analysis of CHD biomarkers

**FIGURE 7:**
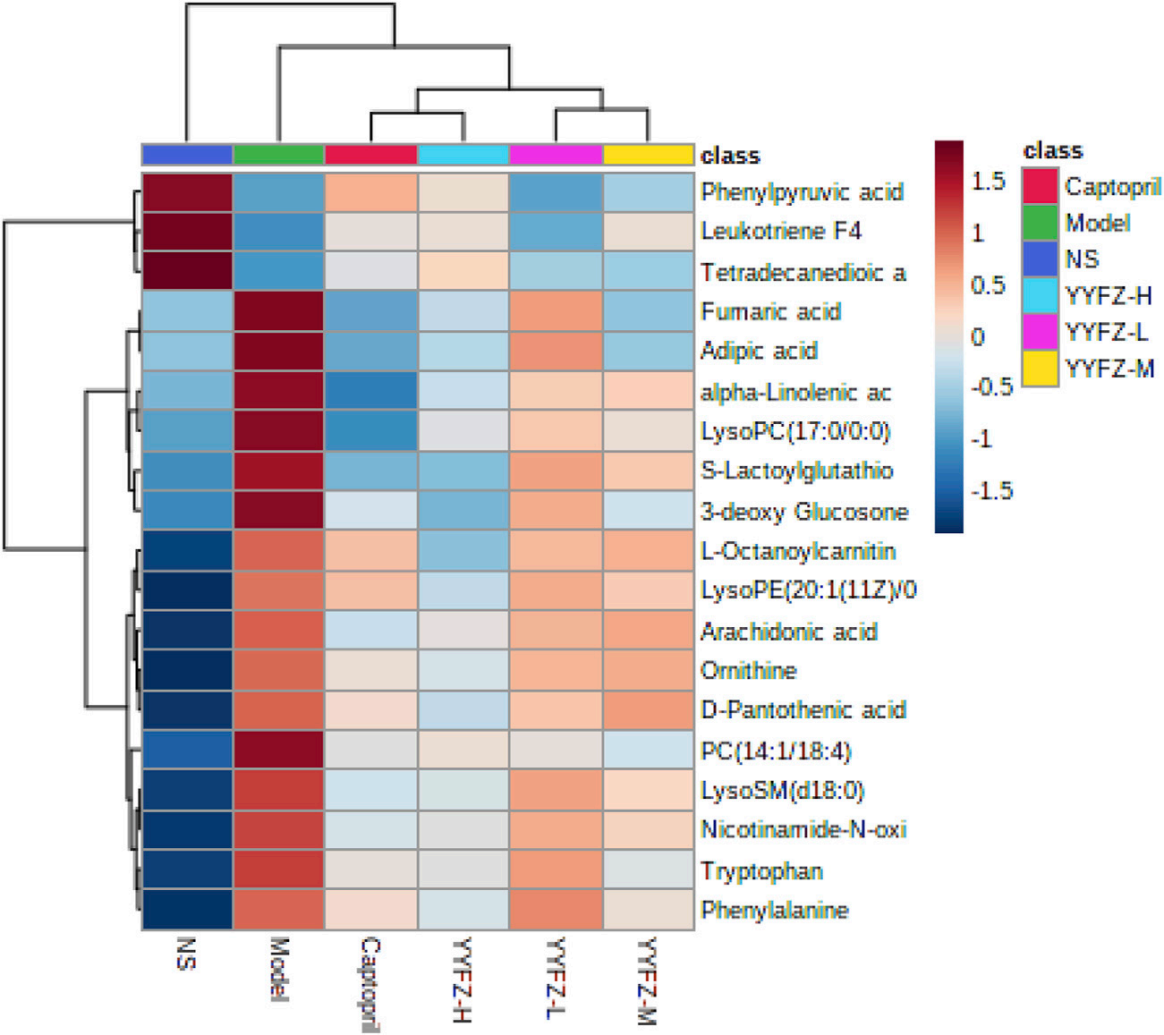
Heat map of serum metabolite changes from minimum (dark blue) to maximum (dark red) in NS, Model, YYFZ-H, YYFZ-M, YYFZ-L and Captopril groups.

To observe the changes of markers in each group more intuitively, this study used hierarchical clustering analysis as shown in Figure 7, which can see the distribution of biomarkers in each group. The shades of color in the figure respond to the magnitude of the values, each row represents a metabolite, and each column represents the content of the whole group of samples; the left bifurcation is the cluster analysis of the substances, where the more clustering levels of the substances indicate the higher similarity of the substances, and there may be similar variations in the source and metabolic pathways. We can observe a significant change in plasma marker content in the Model group compared to the NS group, suggesting that the markers have some discriminatory ability. Metabolite levels in the captopril and YYFZ-H groups were closer to those in the NS group, and metabolite trends were more consistent, suggesting that high-dose YYFZ significantly modulates CHD disease-related markers.

#### 3.2.5 CHD biomarker ROC analysis

ROC curves are widely used for the evaluation of the sensitivity and specificity of markers and can screen for more diagnostic biomarkers. After the above study, we identified markers with diagnostic potential by ROC analysis of the above 19 biomarkers by SPSS 26.0, and the area under the curve was used to evaluate the diagnostic ability of the markers. Usually, an area under the curve>0.5 indicates good discriminatory ability. As shown in Figure 6B, we can see that each curve represents a substance and the area under the curve is distributed between 1 and 0.87 (95% confidence interval), indicating that the 19 markers have the diagnostic ability.

#### 3.2.6 CHD biomarker pathway analysis

To further speculate on the mechanism of CHD metabolic disorders, this study performed metabolic pathway analysis (MetPA) on 19 markers. The MetPA database is a visual metabolic pathway analysis database (www.metaboanalyst.ca), which combines pathway enrichment analysis and topological analysis to assist in screening relevant metabolic pathways. We obtained 16 metabolic pathways (Figure 5B), including phenylalanine, tyrosine and tryptophan biosynthesis, alpha-linolenic acid metabolism, phenylalanine metabolism, and arachidonic acid metabolism are the main metabolic pathways involved, suggesting that lipid metabolism and amino acid metabolism are the pathways to focus on in CHD.

### 3.3 Network pharmacology

#### 3.3.1 YYFZ blood entry components and CHD targets

**FIGURE 8:**
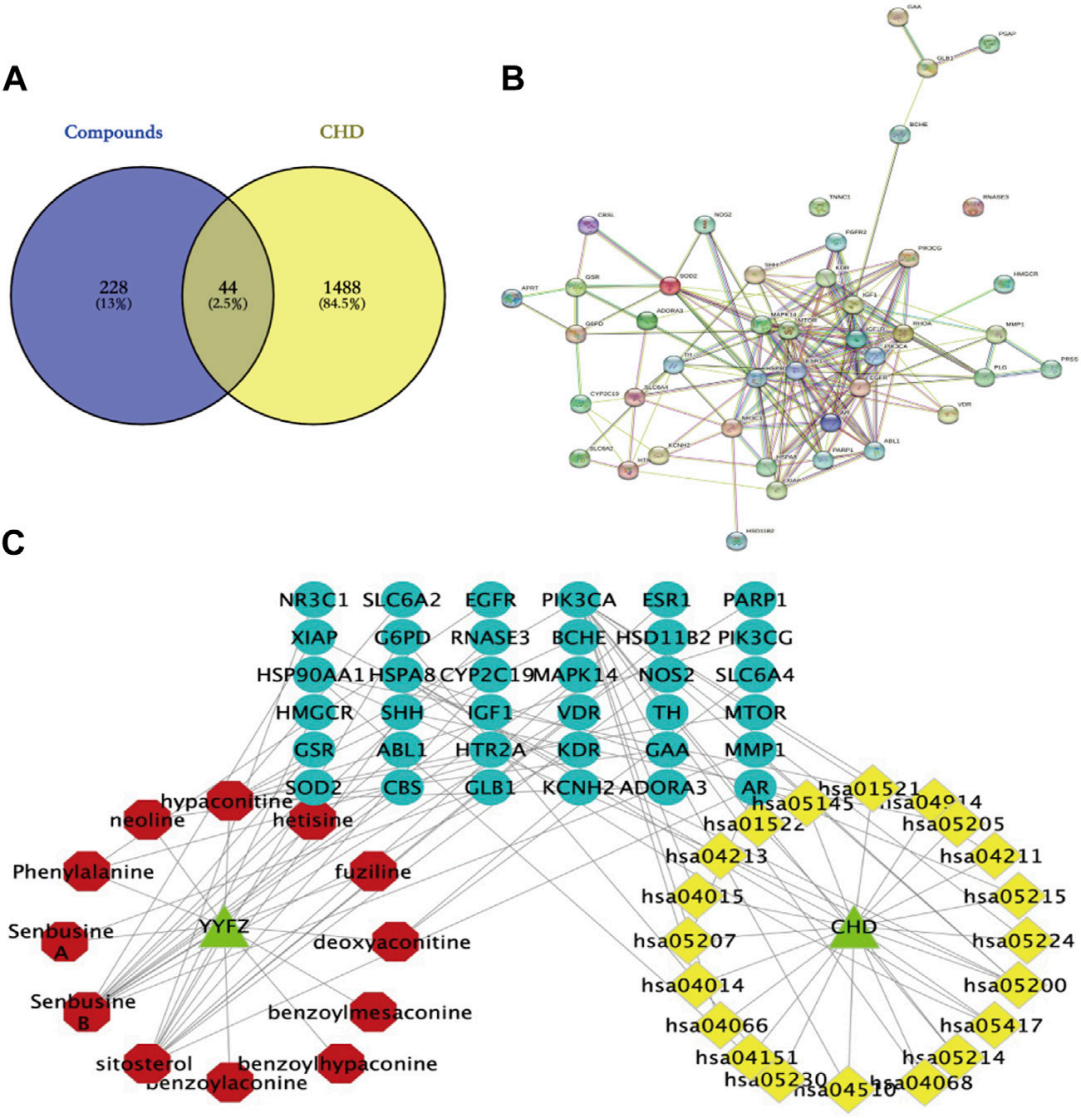
**(A)** Venn diagram of YYFZ component and CHD target; **(B)** PPI diagram; **(C)** Drug-active component-target network expressing the interaction between YYFZ inlet component, protein target and CHD pathway (red-inlet component, blue-target, yellow-pathway).

The database was used to predict the YYFZ inlet component targets, and finally, 228 YYFZ component targets were obtained. 1488 CHD disease targets were obtained. Venny enrichment analysis was performed between CHD inlet component targets and disease targets, as shown in Figure 8A, and 44 YYFZ targets for CHD was obtained.

#### 3.3.2 Visualization and analysis of active ingredient-target-disease network

The potential targets of CHD and YYFZ were imported into the String database for protein interaction analysis and the PPI network was constructed, and the results showed 42 nodes with 176 edges (Figure 8B). The results in.tsv format were imported into Cytoscape 3.8.2 software for network analysis, MAPK14, EGFR, HSP90AA1, MTOR, ESR1, and IGF1 had high Dgree values, which might be the key targets of YYFZ for CHD treatment.

The Cytoscape software was used to construct the drug-active-ingredient-target network (Figure 8C). The network contains 70 nodes and 90 edges, and the larger the node degree value, the more important it is in the network. The core nodes were selected based on the network topological features such as node degree values. Among them, senbusine B, sitosterol, hypaconitine, phenylalanine, and deoxyaconitine may be the pharmacodynamic components of YYFZ for CHD.

**FIGURE 9:**
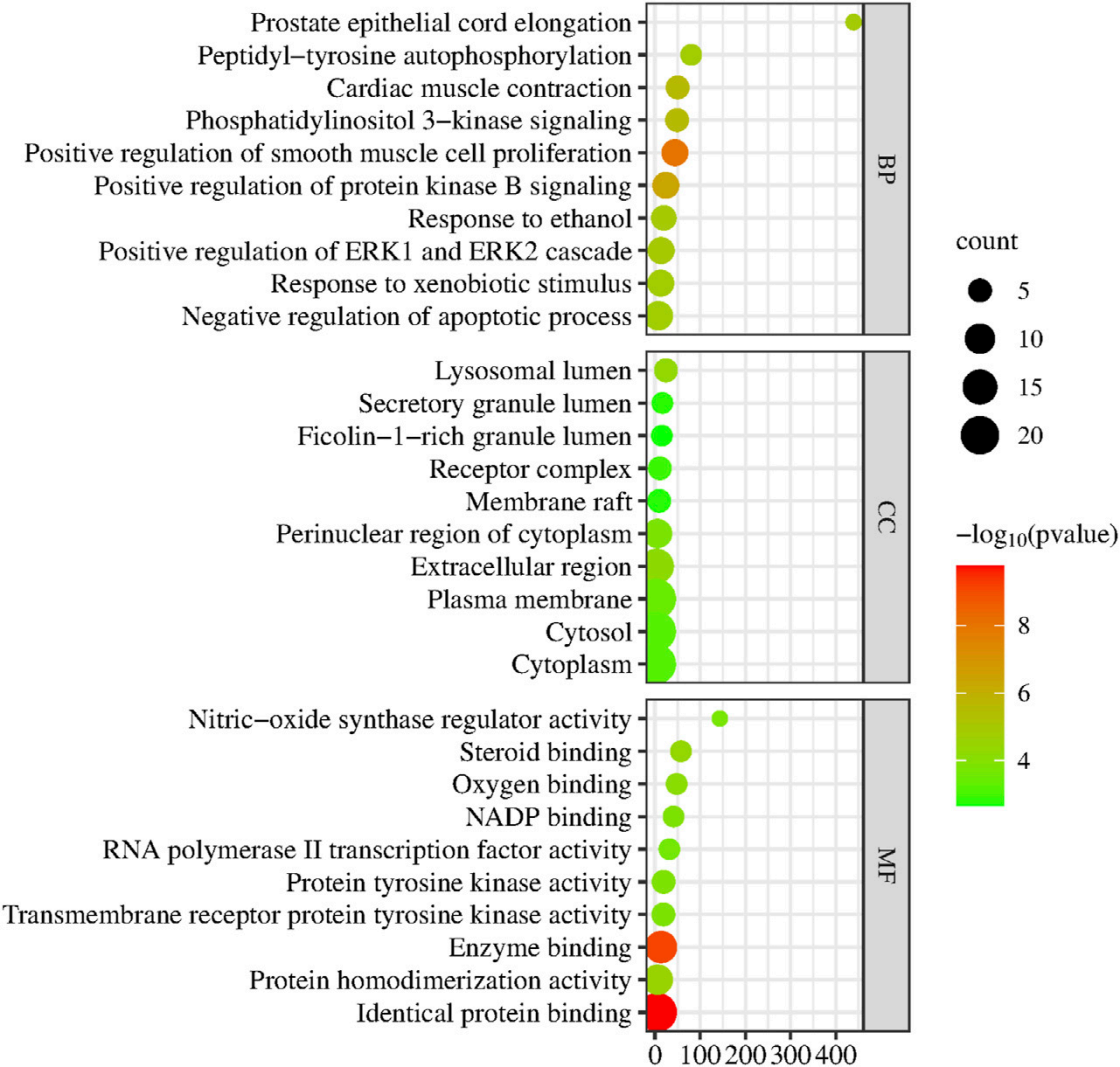
GO function enrichment map.

**FIGURE 10:**
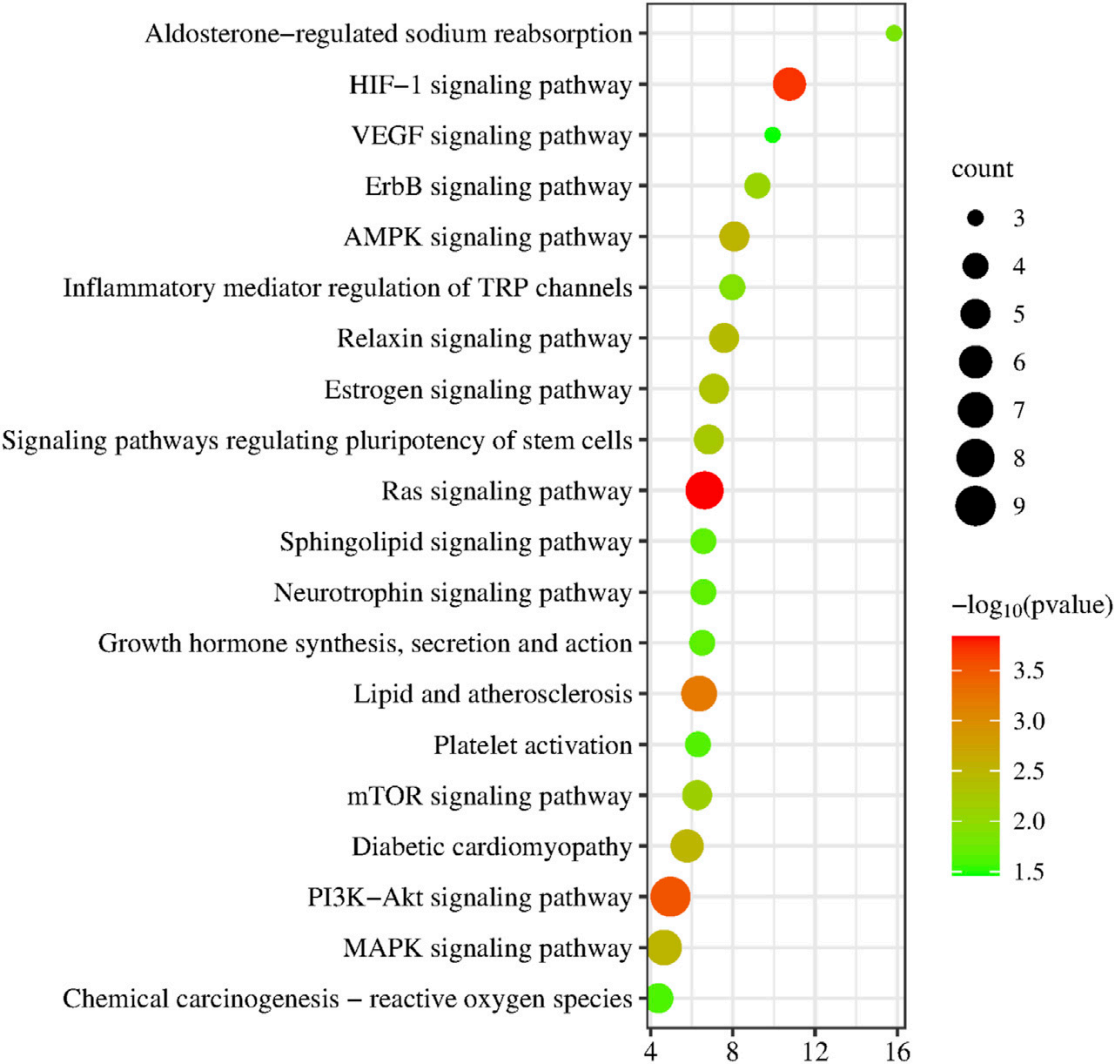
KEGG pathway enrichment analysis.

To further discover the mechanism of action of YYFZ in the treatment of CHD, GO enrichment analysis was performed using the DAVID database for the common targets obtained above (Figure 9), and a total of 162 enrichment results were obtained for biological process (BP) screening at *p* < 0.05, mainly involving positive regulation of protein kinase B signaling, positive regulation of smooth muscle cell proliferation, phosphatidylinositol 3-kinase signaling. Cellular component (CC) obtained 36 enrichment results, mainly involving the extracellular region, lysosomal lumen, perinuclear region of cytoplasm, etc.; molecular function (MF) obtained 37 enrichment results, mainly involving identical protein binding, Enzyme binding, Protein homodimerization activity, etc. The KEGG pathway analysis is shown in Figure 10, excluding the pathways unrelated to CHD. signaling pathway, PI3K-Akt signaling pathway, MAPK signaling pathway, etc.

## 4 Discussion

In this experiment, the CHD model of rats was established by intraperitoneal injection of adriamycin, and the rats were treated with captopril and different doses of YYFZ, and the efficacy of YYFZ was comprehensively evaluated by observing the biochemical indexes and cardiac function indexes of rats and myocardial histopathological sections. Adriamycin belongs to anthracycline antibiotics, which are widely used in clinical practice for the treatment of various malignant tumors (Sabatino et al., 2020; Younis et al., 2021; Xu et al., 2022). Cardiotoxicity is one of its main side effects, which can be manifested as irreversible dose-dependent cardiomyopathy and CHD, so a heart failure model was prepared using this side effect. TNF-*α* is an inflammatory factor that plays an important role in promoting myocardial remodeling and inhibiting myocardial contraction, as well as in increasing endothelial and myocardial apoptosis, and there is abundant evidence that inhibition or reduction has a protective effect in heart failure models (Lee et al., 2019; Reina-Couto et al., 2021; Szabo et al., 2021; Zhong et al., 2022). BNP is a marker of cardiac insufficiency, and many studies have shown that plasma BNP levels are significantly elevated in heart failure patients, which can be used for the diagnosis of heart failure (Ichiki et al., 2013; McDonald et al., 2018; Rørth et al., 2020). We found that the YYFZ-H group had a better therapeutic effect from the perspective of biochemical indexes, significantly regulating serum BNP and TNF-*α* and improving cardiac function (*p* < 0.05). The pathological results showed that the YYFZ dose groups could improve myocardial cell disorder and reduce inflammatory cell infiltration. Echocardiographic determination of cardiac function in rats has been widely accepted because it provides a convenient, reliable, and non-invasive method (Zeng et al., 2019; Quagliariello et al., 2021; Wang X. et al., 2022). Generally, EF is measured as an indicator of cardiac function, FS is also one of the commonly used indicators, and the magnitude of the two values shows a correlation. In the present study, we observed the long axis of the sternum in rats and used EF as an indicator to determine the modeling status of rats and the effect of YYFZ treatment. We found that EF and FS were significantly retraced in the administered group, and there were some differences in the mean values of other functional indicators LVPW;s, LVPW;d, LVID;s, LVID;d, IVS;s, IVS;d, etc. Moreover, the cardiac index of rats in the YYFZ-H and YYFZ-M groups was lower than that of the model group, and ascites were also significantly reduced, suggesting that YYFZ could improve cardiac function and alleviate the symptoms of CHD in rats with CHD.

In this study, we used non-targeted metabolomics techniques to study serum metabolites in CHD rats and identified 19 CHD-related metabolites, seven of which were regulated by YYFZ including S-lactoylglutathione, phenylalanine, fumaric acid, adipic acid, tetradecanedioic acid, *α*-linolenic acid, and lysoPE (20:1(11Z)/0:0), and the metabolic pathways involved mainly include four categories: amino acid metabolism (phenylalanine, tyrosine and tryptophan biosynthesis; phenylalanine metabolism; arginine biosynthesis; alanine, aspartate and glutamate metabolism; arginine and proline metabolism; tryptophan metabolism; tyrosine metabolism), lipid metabolism (*α*-linolenic acid metabolism, arachidonic acid metabolism, biosynthesis of unsaturated fatty acids, glycerophospholipid metabolism), energy metabolism (pyruvate metabolism, pantothenate and CoA biosynthesis, citrate cycle (TCA cycle)), glutathione metabolism.

It has been confirmed that myocardial infarction and heart failure can lead to abnormalities in amino acid metabolism such as branched-chain amino acids, taurine or glutamine, and abnormalities in the metabolism of certain amino acids, such as branched-chain amino acids, can activate the mTOR signaling pathway, which accelerates myocardial remodeling and leads to the development of heart failure after myocardial infarction. In addition, elevated leucine levels may activate mTOR, and inhibition of mTOR in a heart failure model improves cardiac function. When mitochondrial dysfunction and phenylalanine are increased in the heart failure state, tetrahydrobiopterin consumption is increased, resulting in decreased nitric oxide production and causing heart failure (Nishijima et al., 2011; Hong et al., 2019). Phenylalanine may also be associated with increased aromatic amino acids, and increased protein breakdown in muscle and impaired liver function can cause accumulation of aromatic group amino acids. Dysregulation of the hepatic urea cycle can lead to a further increase in ornithine, a precursor to the formation of polyamines. Polyamines are also involved in the development of cardiac disease processes, and in models of myocardial hypertrophy (Chen et al., 2019). This further reflects the regulatory role of amino acid metabolism in heart failure, but the exact mechanisms still need to be investigated in depth (Wang et al., 2016; Dimou et al., 2022).

Disorders of lipid metabolism play an extremely important role in the process of CHD. It has been shown that patients with CHD often have abnormal lipid levels, and that disorders of lipid metabolism further contribute to the development of CHD. In this experiment, linoleic acid was significantly elevated in the model group compared to the blank group, and the molecular structure of linoleic acid contains double bonds that are susceptible to oxidative stress, causing vasodilator dysfunction and endothelial damage (Asselin et al., 2013; Djuricic and Calder, 2021). Phospholipids are the main constituent molecules of biological membrane structure and assist in the function of the organism at the cellular level. Arachidonic acid is a widely distributed unsaturated fatty acid in living organisms, and in pathological states, it is broken down into biologically active derivatives that are involved in inflammatory responses, apoptosis, and other biological processes (Sonnweber et al., 2018). Prostaglandins (PGs) are produced by the metabolism of arachidonic acid through the COX pathway and are further metabolized to produce a variety of active substances that regulate the process of vascular remodeling and generation and can control vascular tone and influence the course of CHD (Mitchell et al., 2021; Wan et al., 2021).

The heart is the organ of the body with the greatest energy demand, and the diastolic and contractile movements of the heart muscle depend on the energy produced by the cells. Under normal conditions, 60%–80% of the heart’s energy is derived from fatty acid oxidation and 10%–20% from glucose metabolism. In contrast, when cardiac function is abnormal, the metabolic substrate is changed from glucose to fatty acids, resulting in increased levels of free fatty acids in serum and myocardial tissue. It has been shown that lipid deposition is observed in rat myocardium high in free fatty acids and that free fatty acids may contribute to impaired cardiac function via PI3K-Akt-GLUT4 and AMPK-eNOS pathways (Han et al., 2018). Acylcarnitine is mainly found in muscle tissues such as cardiac muscle and is involved in fatty acid metabolism and amino acid metabolism. Short-chain acylcarnitines are associated with branched-chain and aromatic amino acid metabolism, and the buildup of long-chain acylcarnitines results from impaired fatty acid oxidation (Roussel et al., 2015). One study found that acylcarnitine decreased in a guinea pig model of compensatory hypertrophy, while fatty acids gradually increased during the process. Long-chain acyl CoA dehydrogenase knockout mice were cardiac hypertrophy accompanied by elevated triglyceride levels, and after carnitine administration, triglyceride levels returned to normal in the myocardium of long-chain acyl CoA dehydrogenase knockout mice (Foster et al., 2016). The above suggests that amino acid metabolism, lipid metabolism and energy metabolism are closely related to the pathogenesis of CHD and that YYFZ acts to inhibit inflammation and reduce intravascular lipid accumulation by regressing lipid and amino acid metabolism.

Hypaconitine, deoxyaconitine and senbusine B are all diterpenoid diester alkaloids with analgesic, anti-inflammatory and antipyretic effects (Zhang et al., 2022). YYFZ is composed of aconitum and Coicis seed. Aconitoid alkaloids in aconitum aconitine are the main active components of YYFZ, while aconitine alkaloids can be hydrolyzed under heating conditions. It is speculated that hypaconitine, deoxyaconitine and senbusine B are the hydrolyzed alkaloids that play a role. Sitosterol is the most abundant sterol in Coix seed, which can interfere with a variety of cell signaling pathways, including cell cycle, cell apoptosis, survival, invasion, proliferation, metastasis, anti-inflammatory, angiogenesis, and cardiac protection (Khan et al., 2022).MAPK14 (p38α), a member of the mitogen-activated protein kinase (MAPK) family, is the most abundant and well-characterized of the four isoforms of p38MAPK and plays a central role in the initiation of stress-activated pro-inflammatory responses (Cuenda and Rousseau, 2007). p38MAPK14 acts downstream on transcription factors and protein kinases to regulate cellular stress and inflammatory responses. It has been demonstrated that p38MAPK plays an important role in myocarditis and myocardial remodeling (Niu et al., 2017; Turner and Blythe, 2019). EGFR is a receptor-type tyrosine kinase, which is important for embryonic heart development and maintenance of adult heart function. EGFR signaling pathway is a complex network system, including three main pathways: Ras/Raf/MAPK pathway, PI3K/AKT pathway, JAK pathway, and STAT pathway (Iwamoto and Mekada, 2006). mTOR is an atypical serine/threonine protein kinase, which can participate in gene transcription and protein expression by phosphorylating its downstream target proteins, and then affect biological activities such as apoptosis. mTOR signaling pathway is one of the effective ways to treat heart failure (Shi et al., 2020). IGF1 is a key hormone that regulates the growth of cardiomyocytes and physiological cardiac hypertrophy. IGF1 is closely related to cardiac hypertrophy and heart failure. In cardiomyocytes, after IGF1 binds to its receptor, the receptor itself is phosphorylated and further activates the MAPK pathway and PI3K pathway (Tao et al., 2017). The Ras family of small guanosine triphosphate (GTP)-binding proteins (G proteins) is one of the major components of intracellular signaling required for normal heart growth and also plays a key role in the development of heart failure. Ras regulates multiple downstream signaling pathways mainly involving MAPK as well as the PI3K/Akt/mTOR pathway (Ramos-Kuri et al., 2021). Overactivation of the JNK and p38 signaling pathways play a key role in the CHD Ras can activate the JNK and p38 signaling pathways through the PI3K/AKT/mTOR pathway. The PI3K-Akt signaling pathway has an important role in the pathogenesis of heart disease, not only regulating the survival and function of cardiomyocytes, but also influencing the proliferation, migration, and apoptosis of vascular smooth muscle cells through the regulation of the pathogenesis and development of CHD (Magaye et al., 2021; Qin et al., 2021; Sun G. et al., 2021). These studies suggest that Ras/MEK/ERK and PI3K/Akt/mTOR signaling control the interconnection of several levels of protein synthesis, cardiomyocyte growth, and remodeling. Ras and its downstream pathways (MAPK pathway, PI3K pathway) may be important pathways for CHD treatment by YYFZ.

## 5 Conclusion

In this study, the pharmacological effects and pharmacodynamic mechanisms of YYFZ in the treatment of adriamycin-induced CHD rats were investigated based on metabolomics combined with network pharmacology. The YYFZ group was able to improve the disorder of cardiomyocyte arrangement and reduce inflammatory cell infiltration, and the cardiac index of rats in the YYFZ-H and YYFZ-M groups was lower than that of the Model group, suggesting that YYFZ was able to improve the cardiac function and alleviate the symptoms of CHD in CHD rats. Metabolomics identified 19 biomarkers related to the pathogenesis of CHD, mainly involving the improvement of amino acid metabolic pathways, fatty acid metabolism, and energy metabolism. In addition, network pharmacology analysis showed that YYFZ may act through MAPK14, EGFR, HSP90AA1, MTOR, ESR1, IGF1, and other protein targets in the treatment of CHD. Combining the results of metabolomics and network pharmacology pathway enrichment analysis suggested that YYFZ may further regulate PI3K/Akt pathway and MAPK pathway by modulating amino acid metabolism and fatty acid metabolism. YYFZ, as a classical TCM formula, has been used to treat CHD from multiple links and targets.

## Data Availability

The raw data supporting the conclusion of this article will be made available by the authors, without undue reservation.
