# Supplementary material for: Pharmacological effects and mechanisms of YiYiFuZi powder in chronic heart disease revealed by metabolomics and network pharmacology
Source: Front Mol Biosci. 2023 Jun 23;10:1203208. doi: 10.3389/fmolb.2023.1203208 (PMC10327484; doi:10.3389/fmolb.2023.1203208)
Supplement: Supplementary file 1 [file Table1.docx]

*In vivo composition analysis of Yiyifuzi powder*

- 1. **Preparation of YYFZ decoction**

Refer to the manuscript under "2.2".

**1.2 Drug administration and grouping**

After adaptive feeding, 6 rats were randomly divided into blank group and medication group, with 3 rats in each group. The rats in the treatment group were treated with YYFZ decoction (1 mL/100g) by gavage for 5 days, and those in the control group were treated with the same amount of water.

- 1. **Sample pretreatment**

At the end of the 4th day of administration, the rats were fasted for 12 hours without drinking water. A final gavage was performed on day 5, and 1 h after the end of the gavage, all rats were anesthetized with 10% chloral hydrate, and blood was collected from the abdominal aorta and stored in EP tubes. After standing for 30 min, the blood samples were centrifuged at 4500 rpm/min, 4°C for 10 min, and the supernatant was aspirated and stored in a − 80°C refrigerator until use

**1.4 Sample preparation**

Precision sucked 200 μL LYYFZ decoction, added 100 μL methanol, vortexed and mixed, centrifuged at 4°C, 13000 rpm/min for 15 min, took the supernatant and dried with nitrogen, then added 200 μl methanol to redissolve, vortexed for 1 min, at 4°C, After centrifugation at 13000 rpm/min for 15 min, the supernatant was removed and prepared for subsequent analysis. After the serum samples stored in the -80°C refrigerator were removed and thawed, 1000 μL of serum was precisely taken, 200 μL of methanol was added to vortex and mixed, centrifuged at 4°C, 13000 rpm/min for 15 min, the supernatant was dried with nitrogen, and then 100 μL of methanol was added to redissolve. The mixture was vortexed for 2 min and centrifuged at 13000 rpm/min for 15 min at 4°C to remove the supernatant for subsequent analysis.

**1.5 Liquid phase condition**

A Waters ACQUITY UPLC BEH C18 column (2.1 mm×100 mm, 1.7 μm) was used. The column temperature was set at 35°C, the flow rate was 0.3 mL/min, and the injection volume was 10 μL. Mobile phase A (0.1% formic acid aqueous solution) -B (0.1% formic acid acetonitrile solution), gradient elution. The gradient elution procedure was as follows: 0-1 min, 1%B; 1-2 min, 1%-5% B; 2-5 min, 5%-15% B; 5-10 min, 15%-30%B; 10-15 min, 30%-50% B; 15-20 min, 50%-70%B; 20-24min,70%-90%.

**1.6 Mass spectrum conditions**

The analysis was performed with an electrospray ionization source (ESI source) in positive ion ionization mode, and high-purity liquid nitrogen was used as the gas for spray ionization and desolvation. The scanning range of MS spectrum was m/z 50-1000. The capillary voltage was 3.0 kV. The desiccator temperature was 325°C; The drying gas flow rate was 10 mL/min; Desolvation gas flow rate (N2) 600 L/h; The ion source temperature was 120°C; Desolvation gas temperature 350°C; The gas flow rate of the cone hole was 50 L/h, and the scanning range was 50-2000.

**1.7 Data processing**

The UPLC-Q-TOF/MS data of YYFZ decoction and serum samples were detected and extracted by MassLynx V 4.1 software (Waters, Manchester, UK). The chemical composition information of YYFZ obtained by literature research was compared, and the compounds were identified by ion fragmentation

**2 Results**

The absorption of drug components in the human body is closely related to the properties of the components themselves. By comparing the serum samples of the blank group and the administration group, as well as the information of quasimolecular ion peaks and secondary mass spectrometry fragments, a total of 12 prototype blood components were identified in the serum of the treated rats. The specific information and configuration are as follows ( Table S1). Ten of the 12 blood components were alkaloids Senbusine B, Senbusine A, hetisine, fuziline, neoline, benzoylmesaconine, benzoylaconine, benzoylhypaconine, hypaconitine, deoxyaconitine; One was the amino acid Phenylalanine and one was the sterol sitosterol. In vitro, we found that alkaloids were the main components of YYFZ decoction, among which mesaconine, benzoylaconine, benzoylmesaconine and benzoylhypaconine were the most abundant. However, alkaloids were still the main constituents in vivo, benzoylmesaconine, benzoylaconine and benzoylhypaconine were relatively high, and the specific results were as follows：

**Table S1 YYFZ blood components**

| No. | tR/min | Name | Formula | Parent ion | Measured value | Theoretical value | ppm | Fragments | Resource |
| --- | --- | --- | --- | --- | --- | --- | --- | --- | --- |
| 1 | 2.93 | Phenylalanine | C9H11NO2 | M+H | 166.0883 | 166.0868 | 9.03 | 120.0841 | Fuzi |
| 2 | 4.02 | Senbusine B | C23H37NO6 | M+H | 424.272 | 424.2699 | 4.95 | 406.2463,388.2544,378.2663, 374.2327 | Fuzi |
| 3 | 4.09 | Senbusine A | C23H37NO6 | M+H | 424.2724 | 424.2699 | 5.89 | 406.2650,388.2526,378.2307, 374.2355 | Fuzi |
| 4 | 4.86 | hetisine | C20H27NO3 | M+H | 330.2073 | 330.2069 | 1.21 | 312.2 | Fuzi |
| 5 | 5.42 | fuziline | C24H39NO6 | M+H | 454.2821 | 454.2805 | 3.52 | 436.2719,418.2622,404.2444 | Fuzi |
| 6 | 5.64 | neoline | C24H39NO6 | M+H | 438.2883 | 438.2856 | 6.16 | 420.2788,402.2668,388.2572, 370.2404,356.2261 | Fuzi |
| 7 | 8.72 | 14-Benzoylmesaconine | C31H43NO10 | M+H | 590.2965 | 590.2958 | -1.19 | 572.2874,558.2725,540.2613,526.2451,508.2355 | Fuzi |
| 8 | 9.25 | benzoylaconine | C32H45NO10 | M+H | 604.3141 | 604.3122 | 3.14 | 586.3070,572.2891,554.2800, 540.2614,522.2501,508.2384 | Fuzi |
| 9 | 9.74 | benzoylhypaconine | C31H43NO9 | M+H | 574.3035 | 574.3016 | 3.31 | 542.2789,524.2621,510.2526, 478.2241 | Fuzi |
| 10 | 11.8 | hypaconitine | C33H45NO10 | M+H | 616.3135 | 616.3122 | 2.11 | 556.2963,584.2888,524.2690, 496.2672 | Fuzi |
| 11 | 12.57 | deoxyaconitine | C34H47NO10 | M+H | 630.3286 | 630.3278 | 1.27 | 570.3034,538.2780,510.2828 | Fuzi |
| 12 | 16.2 | sitosterol | C29H50O | M+H | 415.3946 | 415.394 | 1.44 | 397.2864 | Coix seed |
